# Clinicopathological features and individualized treatment of kidney involvement in B-cell lymphoproliferative disorder

**DOI:** 10.3389/fimmu.2022.903315

**Published:** 2022-09-12

**Authors:** Guangyan Nie, Lianqin Sun, Chengning Zhang, Yanggang Yuan, Huijuan Mao, Zhen Wang, Jianyong Li, Suyan Duan, Changying Xing, Bo Zhang

**Affiliations:** ^1^Department of Nephrology, the First Affiliated Hospital of Nanjing Medical University, Nanjing Medical University, Nanjing, China; ^2^Department of Pathology, The First Affiliated Hospital of Nanjing Medical University, Jiangsu Province Hospital, Nanjing, China; ^3^Department of Hematology, The First Affiliated Hospital of Nanjing Medical University, Jiangsu Province Hospital, Nanjing, China; ^4^Department of Nephrology, Pukou Branch of JiangSu Province Hospital (Nanjing Pukou Central Hospital), Nanjing, China

**Keywords:** lymphoproliferative disorders, Waldenström macroglobulinemia/lymphoplasmacytoid lymphoma, chronic lymphocytic leukemia, non-Hodgkin’s lymphomas, monoclonal gammopathy of undetermined significance, kidney involvement

## Abstract

**Background:**

Due to the various clinical and pathological manifestations of kidney involvement in lymphoproliferative disorder (LPD), the whole spectrum of kidney disease in LPD is still unclear, and data on kidney prognosis is scarce.

**Methods:**

We retrospectively reviewed the renal pathology profiles from January 2010 to December 2021, and 28 patients with B-cell LPD combined with intact renal biopsy data were included.

**Results:**

There were 20 men and eight women aging 41 to 79 years at the time of renal biopsy (median age 62 years). According to hematological diagnosis, patients were classified into four groups: chronic lymphocytic leukemia (CLL) (group1, n=7), Waldenström macroglobulinemia/lymphoplasmacytic lymphoma (WM/LPL) (group 2, n=8; WM, n=6; LPL, n=2), Other non-Hodgkin’s lymphomas (NHL) (group3, n=7; diffuse large B-cell lymphoma (DLBCL), n=2; mucosa-associated lymphoid tissue (MALT) lymphoma, n=4; Low grade B-cell lymphoma, n=1), and monoclonal gammopathy of undetermined significance/monoclonal gammopathy of renal significance (MGUS/MGRS) (group 4, n=6). Median serum creatinine (Scr) level was 129 (range,59-956) umol/L. Eight patients (29%) were presented with acute kidney injury (AKI), and five patients (18%) required hemodialysis upon admission. Twenty-three patients (82%) presented with proteinuria (median protein excretion, 2.14 g/d), 11(39%) of whom had the nephrotic syndrome. Interstitial malignant infiltration was the most frequent renal lesion (n=6). Eight patients underwent immunohistochemistry of renal tissues, of which three patients (CLL, n=1; LPL, n=1; WM, n=1) had confirmed lymphoma infiltrates, and the infiltrating cells in the remaining five patients (CLL, n=1; MALT lymphoma, n=2; MGUS, n=2) were considered unrelated to lymphoma. The most common glomerular diseases were renal amyloidosis (n=4) and membranous nephropathy (n=4). Only 20 patients were treated, 13 of whom were treated with rituximab separately or in combination. The median follow-up time was 11 months. Of these, six had achieved hematological response, complete response in five cases. Eight had achieved renal response. At the end-of-study visit, four patients died and two progressed to end stage kidney disease (ESKD).

**Conclusion:**

In conclusion, the clinicopathological spectrum of renal involvement in BLPD is diverse. Renal biopsy and immunohistochemistry are required for early diagnosis and prognostic assessment.

## Introduction

Chronic B-cell lymphoproliferative disorder (B-CLPD) is a group of mature B-cell clonal proliferative disorders characterized by clonal proliferation of mature lymphocytes in the peripheral blood/bone marrow and diagnosed by morphological, immunophenotypic and cytogenetic features of the peripheral blood/bone marrow. It includes chronic lymphocytic leukemia (CLL), multiple myeloma (MM), Waldenström macroglobulinemia/lymphoplasmacytic lymphoma (WM/LPL), and other non-Hodgkin’s lymphomas (NHL) (1). Kidney damage is a common and severe complication of B-CLPD, which often manifests as proteinuria, hematuria, nephrotic syndrome or acute kidney injury (AKI). The direct causes of AKI in B-CLPD include tumor cell infiltration, hypercalcemia, tumor lysis syndrome, and urinary tract obstruction due to lymph node enlargement (2). Moreover, the prevalence of AKI also has an association with the side effects of tumor-targeting treatment such as infection and chemotherapy toxicity. Another important presentation of LPD-related kidney dysfunction is monoclonal immunoglobulin-associated kidney injury, including light chain amyloidosis (AL), light chain deposition disease (LCDD), cryoglobulinemic glomerulonephritis, proliferative glomerulonephritis with monoclonal immunoglobulin deposition (PGNMID), immunotactoid glomerulonephritis, cast nephropathy, etc. The amino acid sequence and physicochemical properties of the variable regions of the pathogenic heavy and/or light chains may be major determinants of nephrotoxicity which could determine the deposition pattern in the kidney (2). However, the potential underlying mechanisms have not been elucidated clearly.

Majority of the kidney involvement in LPD were associated with pathogenic monoclonal immunoglobulin which were mainly observed in patients with indolent B-cell lymphoma or monoclonal gammopathy of undetermined significance (MGUS). The physicochemical properties of the secreted monoclonal immunoglobulin (MIg) instead of tumor masses lead to the disorders (3). Recently the term of monoclonal gammopathy of renal significance (MGRS) has been suggested when an MGUS is causing kidney disease in the absence of a malignancy warranting treatment (4). It is a new classification of pathogenic clonal proliferative disorders that produce a nephrotoxic protein, the diagnosis of which can be established only by performing a kidney biopsy (5). In addition to MIg deposition, there may be other mechanisms involved in the secretion of various biologic factors and/or MIg autoantibody activity in MGRS. For example, secreting vascular endothelial growth factor in polyneuropathy, organomegaly, endocrinopathy, M-protein, skin changes (POEMS) syndrome may trigger the development of renal endothelial cell injury and thrombotic microangiopathy (TMA) (6), or membrane proliferative glomerulonephritis (MPGN) which were induced by a dimeric monoclonal lambda light chain (LC) as a “micro autoantibody” to complement factor H (7). A diversity of disease manifestation has been reported in MGRS, from isolated proteinuria to the development of end stage kidney disease (ESKD), which therefore require timely clone-directed treatment targeting the B-cell monoclonal proliferation responsible for the production of nephrotoxic Ig or Ig subunits (8).

Due to the various clinical and pathological manifestations of kidney involvement in LPD, it is difficult for clinical diagnosis and subsequent treatment, and therefore the prognosis is unsatisfactory (9, 10). The whole spectrum of kidney disease in LPD is still unclear, and data on kidney prognosis is scarce. We herein retrospectively analyzed the spectrum of kidney damage in patients with LPD which were confirmed by renal biopsy to shed light on timely diagnosis and individualized therapy in LPD-related renal damage.

## Materials and methods

### Subjects

**Figure 1:**
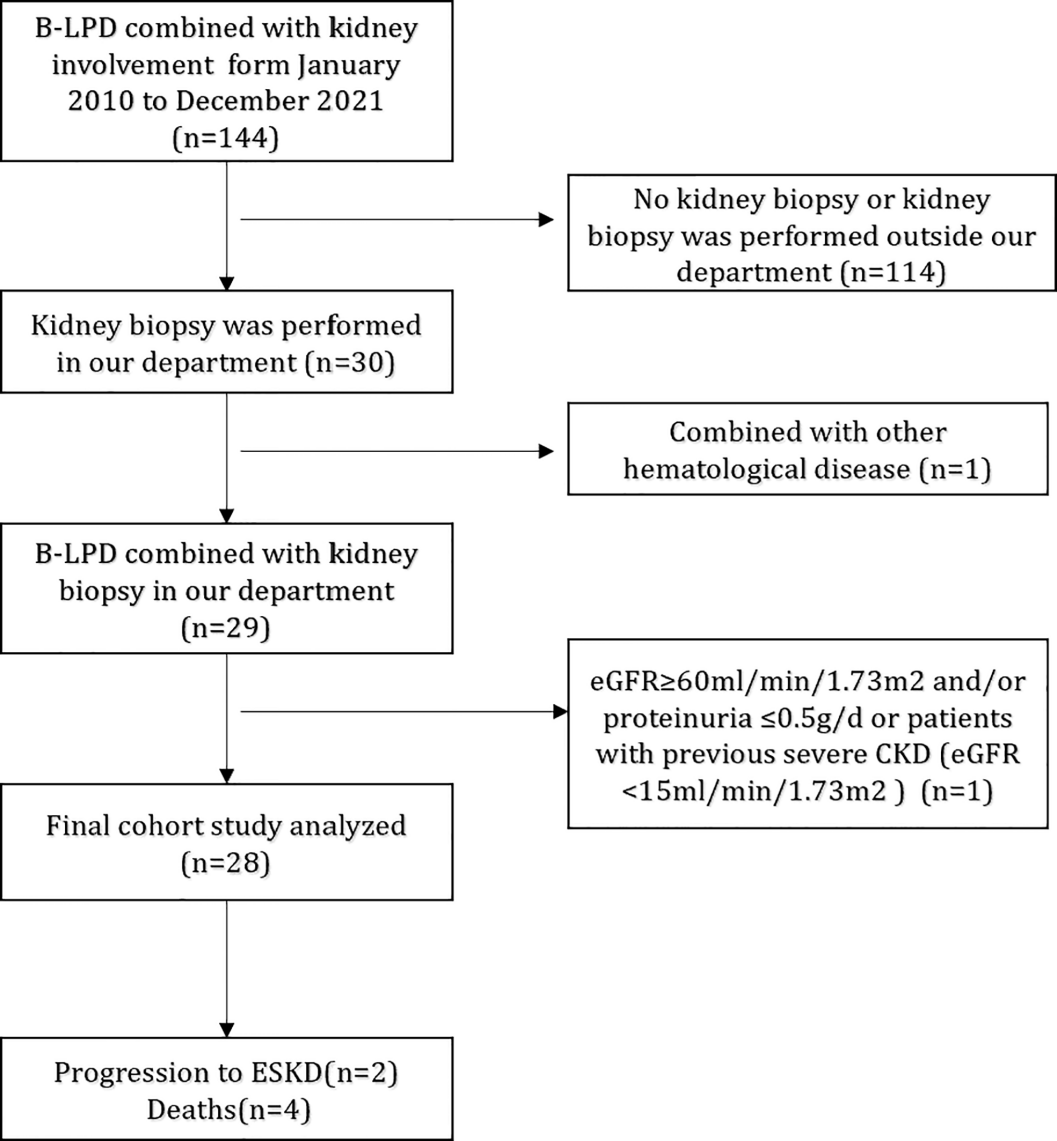
Flowchart of study participants. B-LPD, B-cell lymphoproliferative disorders; eGFR, estimated glomerular filtration rate; CKD, chronic kidney disease; ESRD, end-stage renal disease.

We retrospectively reviewed the renal pathology profiles at the renal department of the First Affiliated Hospital of Nanjing Medical University from January 2010 to December 2021, and 28 patients with B-LPD combined with intact renal biopsy data were included (**Figure 1**). Diagnosis of B-LPD is based on the World Health Organization classification revised in 2016 (11). Inclusion criteria were as follows: (i). B-LPD are clearly diagnosed; (ii). Renal biopsy pathological examination was performed after excluding contraindications;(Iii). The estimated glomerular filtration rate (eGFR) <60ml/min/1.73m2 and/or proteinuria >0.5g/d; (iv). Intact baseline clinical, pathological and laboratory data. Exclusion criteria: (i). eGFR <15ml/min/1.73m2; (ii)Combined with other tumors or hematological disease. This study was approved by the Ethics Committee of the First Affiliated Hospital of Nanjing Medical University.

### Hematological and immunologic studies

All patients underwent a complete clinical and laboratory workup, including a thorough hematological examination, serum and urine protein electrophoresis, immunochemical characterization of the monoclonal component, and a bone marrow biopsy with aspiration. Conventional cytogenetic analysis was performed as part of the clinical workup for bone marrow aspirate with standard methods in the clinical laboratories. Mutations in myeloid differentiation primary response 88 (MYD88) whole exons were assessed on bone marrow aspirate or peripheral blood samples using the previously-described allele-specific polymerase chain reaction (12) or the Sanger sequencing (13). Immunophenotypic analysis of renal infiltration was available for eight patients. International consensus criteria were used for the diagnosis of CLL, WM/LPL, MGUS/MGRS, and other NHL (4, 14, 15). Hematological response was defined according to the international response evaluation criteria for B-LPD (16–19).

### Renal diagnostic criteria and definitions of renal response

eGFR was calculated using the CKD-EPI (Chronic Kidney Disease Epidemiology Collaboration) creatinine equation (20). Nephrotic syndrome was defined as 3.0 g/day of proteinuria, hypoalbuminemia (<3.0 g/dl), with hyperlipidemia and oedema. The Acute Kidney Injury Network (AKIN) consensus criteria were used to diagnose AKI (21). Chronic kidney disease (CKD) stages were determined using the Kidney Disease Improving Global Outcomes (KDIGO) guideline (22). Renal response was assessed using the following definitions: complete response by achievement of eGFR >60 ml/min per 1.73 m^2^ and proteinuria <0.5 g/d; partial response by a ≥50% increase in eGFR or reduction in proteinuria by 50% with stable renal function for patients with baseline eGFR >60 ml/min per 1.73m^2^. ESKD was defined as the initiation of maintenance dialysis or kidney transplantation.

### Pathologic studies

All kidney biopsy samples were subjected to light microscopy, immunofluorescence microscopy and electron microscopy (EM). Samples were parceled and sectioned at 2 mm, followed by periodic acid-Schiff (PAS), Masson or periodic acid-silver methylamine (PASM) staining. The slices were stained systematically with Congo red and checked under polarized light. For immunofluorescence, frozen sections of 3 mm were stained for ɑ, γ, μ heavy chain, κ and λ light chain (Dakopatts, Glostrup, Denmark), C3, C4 and C1q (Morphosys AbD, Düsseldorf, Germany) by using polyclonal isothiocyanate fluorophore conjugates. Bone marrow smears or biopsies were performed on all patients. Immunohistochemical studies were performed on kidney and bone marrow biopsy samples using anti-CD20, CD19, CD3 and anti-CD38 antibodies (Dakopatts, Glostrup, Denmark).

## Results

### Clinical findings

The clinical characteristics of all 28 patients were summarized in **Table 1**. There were 20 men and eight women aging 41 to 79 years at the time of renal biopsy (median age 62 years). Among them, 13 (46%) of 28 patients had an established diagnosis of B-LPD, the remaining 15 patients had renal biopsies performed almost simultaneously with the diagnosis of B-LPD. 16 (57%), six (21%), and two (7%) patients had a history of hypertension, diabetes mellitus, and hepatitis B respectively. Only one patient (4%) had previously known stage II chronic kidney disease (CKD).

**Table 1:** Baseline and demographic characteristics at the time of kidney biopsy.

	All (n=28)	CLL (n=7)	WM/LPL (n=8)	Other-NHL (n=7)	MGUS/MGRS (n=6)
Demographic characteristics					
Age,yr [range]	62.5 [41-79]	66 [45-79]	64.5 [53-73]	58 [41-67]	66.5 [54-74]
Male, %	20/28 (71%)	5/7 (71%)	8/8 (100%)	3/7 (43%)	4/6 (67%)
Comorbid conditions					
Hypertension	16/28 (57%)	2/7 (29%)	6/8 (75%)	4/7 (57%)	4/6 (67%)
Diabetes mellitus	6 /28 (21%)	0	2/8 (25%)	1/7 (14%)	3/6 (50%)
Hepatitis B	2 /28 (7%)	0	0	2/7 (29%)	0
History of CKD	1 /28 (4%)	0	0	0	1/6 (17%)
Laboratory data					
Hemoglobin, g/L	102 [66-143]	102 [82-133]	93 [73-135]	100 [66-143]	98 [81-108]
White blood cell, *10^9/L	7.15 [1.74-51.54]	20.20 [3.80-51.50]	5.78 [4.90-21.60]	6.82 [1.74-9.24]	7.14 [4.71-9.07]
blood platelet, *10^9/L	166 [82-424]	135 [98-187]	158 [82-424]	250 [86-319]	202 [118-272]
Lymphocyte count, *10^9/L	1.50 [0.39-45.67]	10.60 [1.49-45.70]	1.66 [0.56-20.20]	1.32 [0.39-1.69]	1.02 [0.59-1.70]
Serum albumin, g/L	32.3 [14.9-47.8]	33.5 [20.6-47.8]	31.2 [14.9-41.0]	30.3 [15.3-34.5]	33.9 [17.5-41.8]
Serum globulin, g/L	24.2 [13.2-68.3]	23.5 [18.7-46.2]	34.4 [13.2-68.3]	26.9 [18.8-40.4]	23.5 [18.0-34.9]
Serum creatinine, umol/l	129 [59-956]	134 [59-790]	135 [72-425]	83 [68-189]	231 [73-956]
Serum urea, mmol/l	8.98 [2.90-20.67]	8.15 [5.22-20.70]	11.30 [4.02-15.30]	5.71 [2.90-9.00]	13.10 [6.93-19.80]
eGFR, mL/min/1.73 m2	48 [4-106]	44 [7-99]	46 [13-106]	71 [31-97]	24 [4-73]
Hematuria	21 [2-1543]	22 [4-968]	22 [4-175]	44 [13-1543]	8 [2-14]
Proteinuria, g/d	2.14 [0.06-21.22]	1.36 [0.06-4.22]	3.38 [2.04-21.20]	1.13 [0.35-19.30]	2.81 [0.20-19.80]
Nephrotic syndrome	11/28 (39%)	2/7 (29%)	4/8 (50%)	3/7 (43%)	2/6 (33%)
Acute kidney injury	8/28 (29%)	2/7 (29%)	3/8 (38%)	0	3/6 (50%)
Hemodialysis	5/28 (18%)	1/7 (14%)	1/8 (13%)	0	3/6 (50%)
sIgM,g/L	0.93 [0.29-48.50]	0.69 [0.56-0.92]	6.46 [0.57-48.50]	1.06 [0.61-5.76]	0.80 [0.29-10.80]
sIgG, g/L	8.0 [1.5-48.4]	10.6 [4.9-24.7]	4.2 [1.5-48.4]	8.1 [3.1-16.9]	5.9 [3.4-10.0]
sIgA, g/L	1.48 [0.12-5.77]	1.48 [0.84-2.55]	0.48 [0.12-1.35]	2.56 [0.87-4.19]	1.63 [0.76-5.77]
Autoantibodies					
ACA	3/22 (14%)	0	2/6 (33%)	1/7 (14%)	0
ANA	3/27 (11%)	1/7 (14%)	0	2/7 (29%)	0
Anti-PLA2R antibody	1/21 (5%)	0	0	0	1/5 (20%)
Serum monoclonal Ig					
SIFE	/	IgGκ:1/4	IgMκ:4/7 IgMλ:2/7	0	IgMλ:2/6 IgGλ:1/6 IgAκ:2/6λLC:1/6
Abnormal κ/λ ratio	1.64 [0.18-24.10]	2.09 [1.58-2.92]	3.28 [0.18-24.10]	1.45 [1.18-2.03]	1.57 [0.31-2.64]
Morphological abnormalities					
Kidney enlargement	1/28 (4%)	1/7 (14%)	0	0	0
Splenomegaly	13/28 (46%)	5/7 (71%)	4/8 (50%)	2/7 (29%)	2/6 (33%)
Lymphadenopathy	15/26 (58%)	6/7 (86%)	5/7 (71%)	3/6 (50%)	1/6 (17%)
Serum complement level					
sC3, g/L	0.82 [0.25-1.73]	1.02 [0.50-1.09]	0.59 [0.25-1.04]	1.07 [0.40-1.73]	0.80 [0.70-1.09]
sC4, g/L	0.251[0.015-0.700]	0.260 [0.015-0.347]	0.147 [0.067-0.292]	0.291 [0.033-0.700]	0.262 [0.184-0.480]

CKD, chronic kidney disease; eGFR, estimated glomerular filtration rate; ACA: anticardiolipin antibody; ANA, antinuclear antibody; Anti-PLA2R antibody, Anti-phospholipase A2 receptor antibodies; SIFE, serum immunofixation electrophoresis.

Unless otherwise indicated, values for categorical variables are given as number or number/number analyzed (percentage); values for continuous variables, as median [range].

### Hematological and immunologic studies

Bone marrow biopsy was performed in all patients. According to hematological diagnosis, patients were classified into four groups: CLL (group1, n=7), WM/LPL (group 2, n=8; WM, n=6; LPL, n=2), Other-NHL (group3, n=7; DLBCL, n=2; =2; mucosa-associated lymphoid tissue (MALT) lymphoma, n=4; Low grade B-cell lymphoma, n=1), and MGUS/MGRS (group 4, n=6) (**Table 1**). The anatomic sites of sampling used for the primary diagnosis of the lymphoid neoplasm were the lymph nodes (n = 4), bone marrow (n = 13), stomach (n = 3), liver (n = 1), and parotid gland (n = 1).

Four of the eight WM/LPL patients were detected positive for the myeloid differentiation primary response 88 (MYD88) mutation by genetic testing. Recently the MYD88 mutation has been emerged as a hallmark of WM/LPL, implicating in its diagnosis, management, and treatment (23).1 patient with LPL was diagnosed with IgG4-associated disease 7 years after the diagnosis of hematological disease.

Of 28 patients with available data, 15 had an apparently indolent disease without infiltration of hematopoietic tissues. The median serum light chain ratio was 1.64 (range, 0.18-24.10). Among nine patients testing with free light chains, all had abnormal free light chain ratios. In 13 patients (46%), the hematological diagnosis preceded renal manifestations, within a mean time of 36 months. Of these, four had received chemotherapy at the 21, 42, 44, and 83 months, respectively, before the diagnosis of kidney disease. Attention needs to be paid to a patient whose kidney disease was diagnosed 3 months before the diagnosis of hematological disease.

Serum immunofixation electrophoresis was conducted in 22 patients, and a serum MIg was detected in 13 cases (59%). The isotype was IgM (n=8, 36%), IgG (n=2, 9%), and IgA (n=2, 9%) respectively. The LC isotype was κ in 32% of cases.

### Kidney manifestations

At the time of kidney biopsy, median serum creatinine (Scr) level was 129 (range,59-956) umol/L. Eight patients (29%) were presented with AKI, and five patients (18%) required hemodialysis upon admission. Twenty-three patients (82%) presented with proteinuria (median protein excretion, 2.14 g/d), 11(39%) of whom had the nephrotic syndrome. Eight patients (29%) had microhematuria. In the other-NHL group, none were identified with AKI, mainly presenting with proteinuria. Renal ultrasound revealed morphologic abnormalities in one patient (4%), with kidney enlargement (**Table 1**). In another case, the imaging showed speckled hypodense shadow in both kidneys with possible lymphoma infiltration. Extrarenal manifestations (skin purpura) were observed in two patients with positive cryoglobulin.

### Kidney pathological findings

**Figure 2:**
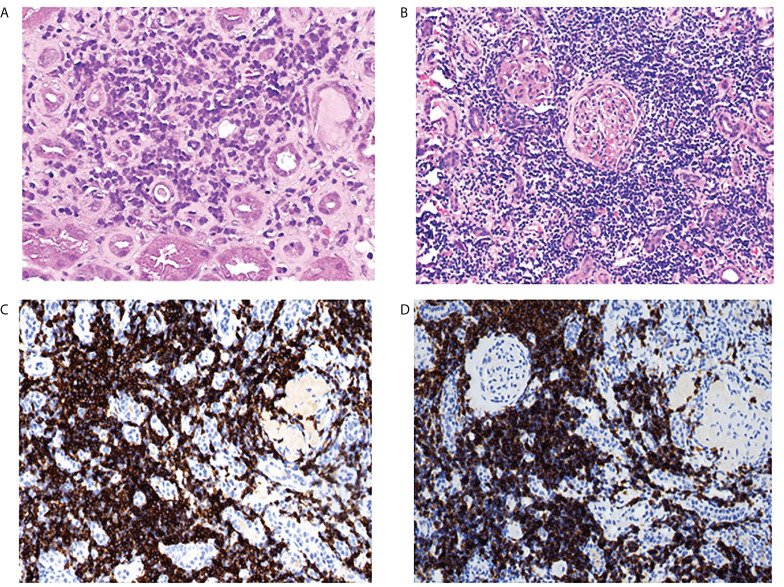
Kidney pathological finding. lymphocytic infiltration of the renal interstitium. **(A)** scattered atypical cells visible in the interstitium in one patient with LPL (PAS,original magnification×400). **(B)** diffuse and massive inflammatory cell infiltration in the interstitium in a patient with CLL (hematoxylin and eosin staining, original magnification×400), the infiltrating cells were positive for CD20 **(C)** and CD23 **(D)** by immunohistochemistry analysis (original magnification×200).

The pathological findings of the renal biopsies are summarized in **Table 2**. Interstitial malignant infiltrates were the most common renal lesion (n=6). Eight patients underwent immunohistochemical studies of renal tissue, of which three patients (CLL, n=1; LPL, n=1; WM, n=1) had confirmed malignant lymphocytic infiltration (**Figure 2**), and the infiltrating cells in the remaining five patients (CLL, n=1; MALT lymphoma, n=2; MGUS, n=2) were considered unrelating to lymphoma. The corresponding clinicopathological findings of the eight patients were shown in **Table S1**.

**Table 2:** Renal biopsy findings.

	All(n=28)	CLL (n=7)	WM/LPL(n=8)	Other-NHL(n=7)	MGUS/MGRS(n=6)
Light microscopy					
Glomeruli	15 [3,59]	13 [7,19]	16 [3,34]	15 [4,59]	16 [7,44]
Sclerotic glomeruli	2 [0,40]	3 [0,7]	2 [0,7]	3 [0,26]	7 [1,40]
Amyloid deposits	4/28	1/7	1/8	1/7	1/6
Mesangial hypercellularity	27/27	6/6,0/4/2/0a	8/8,0/6/1/1a	7/7,0/4/2/1a	6/6,0/3/2/1a
Endocapillary proliferation	5/27	0/6	2/8	2/7	1/6
Crescent formation	8/27	3/6	2/8	3/7	0
Glomerular thrombi	21/27	4/6	7/8	6/7	4/6
Interstitial fibrosis	20/27	2/6,4/1/1/0a	7/8,1/6/1/0a	4/7,3/3/1/0a	5/6,1/3/2/0a
Lymphomatous interstitialinfiltration	6/28	3/7	3/8	0	0
Tubular atrophy	21/27	2/6,4/1/1/0a	8/8,0/6/1/1a	7/7,0/6/0/1a	4/6,2/3/1/0a
Acute tubular necrosis	7/27	3/6,3/3/0/0a	1/8,7/1/0/0a	0	3/6,3/2/1/0
Composition of deposits by immunofluorescence		IgA: 2/7 IgM: 4/7 κLC: 1/7	IgA: 1/8 IgM: 6/8 IgG: 1/8 κLC: 3/8λLC: 2/8	IgA: 5/7 IgM: 6/7 IgG: 5/7 κLC: 2/7	IgA: 3/6 IgM: 4/6 IgG: 2/6 κLC: 2/6
Pathologic diagnosis					
Amyloidosis	4/28	1/7	1/8	1/7	1/6
Cryoglobulinemic GN	2/28	0	1/8	1/7	0
MIDD	1/28	0	1/8	0	0
Membranousglomerulonephritis	4/28	1/7	1/8	1/7	1/6
C3 glomerulonephritis	2/28	1/7	0	0	1/6
TMA	2/28	0	0	0	2/6b
Lymphoma infiltration	6/28	3/7	3/8	0	0
Acute interstitial nephritis	2/28	0	0	0	2/6
IgA nephropathy	2/28	0	0	2/7	0
FSGS	3/28	1/7	1/8	1/7	0
Lupus nephritis	1/28	0	0	1/7	0
Diffuse proliferative sclerosingglomerulonephritis	1/28	0	0	0	1/6

CLL, chronic lymphocytic leukemia; WM/LPL, Waldenström macroglobulinemia/ lymphoplasmacytic lymphoma; NHL, non-Hodgkin's lymphoma; MGUS, monoclonal gammopathy of undetermined significance; MGRS, monoclonal gammopathy of renal significance; MIDD, monoclonal immunoglobulin deposition disease; TMA, thrombotic microangiopathy; FSGS, focal segmental glomerulosclerosis.

a: none/mild/moderate/severe. b: secondary diagnosis.

**Figure 3:**
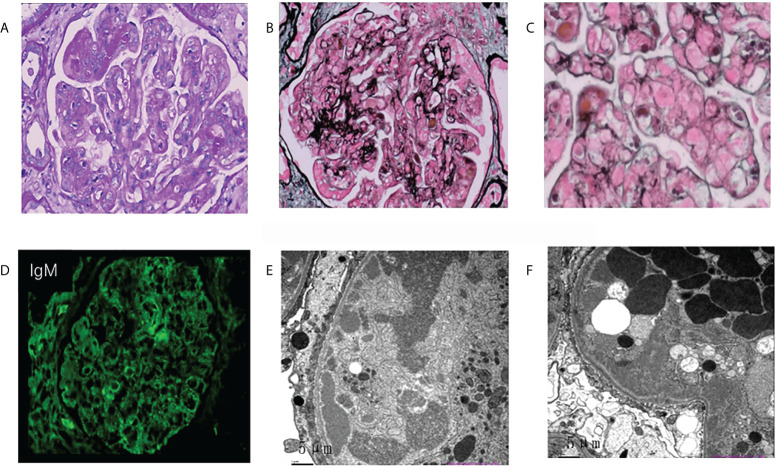
Morphologic features of membranoproliferative glomerulonephritis on renal biopsy of the patient with Waldenstrom’s macroglobulinemia. **(A)** Mesangial cells were severely proliferated and lobulated, and mesangial matrix was moderately increased. (asterisks; PAS, original magnification×400). **(B, C)** Deposits were observed in mesangial, subcutaneous and subepithelial areas (PASM. b, original magnification×400; c, original magnification×600). **(D)** Deposits stained with IgM in the mesangial area and para mesangial area by immunofluorescence (original magnification×400). **(E, F)** ByEM, electron-dense deposits were observed in the subendothelial, mesangial area and medial of basement membrane. Foot processes showed diffuse fusion (original magnification×2500). PAS, periodic acid–Schiff; PASM, periodic acid-silver metheramine; EM, electron microscopy.

**Figure 4:**
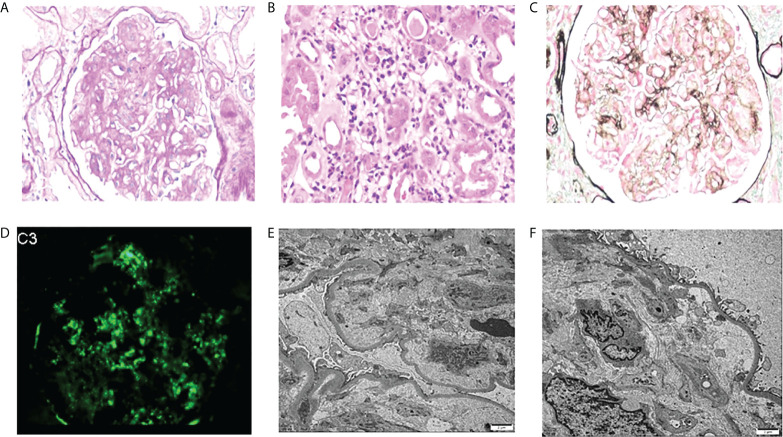
Morphologic features of C3 glomerulonephritis with thrombotic microangiopathy on renal biopsy of the patient with MGRS. **(A)** Mesangial cells were severely proliferated and lobulated (PAS, original magnification×400). **(B)** Diffuse inflammatory cells infiltrate in the renal interstitium (PAS, original magnification×400). **(C)** Endothelial cell proliferation, capillary occlusion, visible microthrombosis, and basement membrane thickening were observed by PASM-Masson (original magnification×400). **(D)** Deposits stained with C3 in the mesangial area by immunofluorescence (original magnification×400). **(E, F)** By EM, TMA-like changes were observed in the glomerulus (original magnification×2500). MGRS, monoclonal gammopathy of renal significance; TMA, thrombotic microangiopathy; monoclonal gammopathy of undetermined significance.

The most common glomerular diseases were renal amyloidosis (n=4; CLL, n=1; WM, n=1; Other NHL, n=1; MGRS, n=1) and membranous nephropathy (n=4; CLL, n=1; WM, n=1; Other NHL, n=1; MGUS, n=1). A patient with MGUS had primary membranous nephropathy with positive serum anti-phospholipase A2 receptor (PLA2R) antibodies and diffuse granular deposits of anti-PLA2R antibodies in the basement membrane detected by immunofluorescence. Two patients were diagnosed with cryoglobulinemic glomerulonephritis (WM, n=1, MALT lymphoma, n=1), one of which was the type I IgM-κ cryoglobulinemic glomerulonephritis. The renal pathology of WM was a typical type of MPGN, with heavy hyperplasia and lobularity of the mesangial cells by light microscopy, and IgM-κ deposition in the mesangial region by immunofluorescence. Electron-dense deposits were found in the subendothelial, mesangial, and basement membrane region by electron microscope (EM) (**Figure 3**). One case having cryoglobulinemic glomerulonephritis combined with MALT lymphoma, glomerular and interstitial mononuclear cell infiltration was observed by light microscopy. One patient of monoclonal immunoglobulin deposition disease (MIDD) combined with WM, displaying IgM-λ deposition in the mesangial region by immunofluorescence. Two patients were diagnosed with C3 glomerulonephritis (CLL=1, MGRS=1), with a combined diagnosis of TMA on renal pathology in the MGRS patient (**Figure 4**). Another patient of MGRS had a combined diagnosis of diffuse proliferative sclerosing glomerulonephritis and TMA. Additional glomerulonephritis unrelated to MIg included, two cases of IgA nephropathy (IgAN) (DLBCL, n=1; MALT lymphoma, n=1), three cases of focal segmental glomerular sclerosis (FSGS) (CLL, n=1; WM, n=1; Other NHL, n=1), and one case of proliferative lupus nephritis occurring in DLBCL.

### Treatments and patient outcomes

8 of the 28 patients did not receive chemotherapy, and two patients with MGUS received methylprednisolone and prednisone/rituximab for acute interstitial nephritis (AIN) and membranous nephropathy, respectively. Of the remaining 18 patients, 10 were receiving rituximab-based therapy regimen and two patients were treated with ibrutinib (**Table 3**). Hematological response was assessable in 13 patients. Of these, six had achieved hematological response, complete in five cases. 16 patients could be evaluated for renal response after therapy. Of these, eight had achieved renal response, complete remission in three cases. In the WM group, complete response was achieved in one patient with membranous nephropathy and partial response was achieved in three patients with acute interstitial nephritis and cryoglobulinemic glomerulonephritis. Three patients who achieved hematological response also confronted with renal response.

**Table 3:** Treatment Regimens and Outcomes.

number	Haematologicaldiagnosis	Diagnosis by renalbiopsy	Treatment Regimens	Hematologicalresponse	Renal response	Follow-uptime (months)	Patientoutcome
1	CLL	Lymphoma infiltration	NT	NA	NA	12.37	Died
2	CLL	FSGS	Ibrutinib	Stable	No	18.97	Alive
3	CLL	AL	Rituximab	No	No	Loss tofollow-up	
4	CLL	MN	FCR	CR	NA	136.47	Alive
5	CLL	AIN	R + Obutinib	No	No	1.67	Alive
6	CLL	Lymphoma infiltration	FC	NA	NA	Loss tofollow-up	
7	CLL	C3 glomerulonephritis	NT	NA	NA	2.97	Alive
8	WM	AIN	RCD	CR	PR	20.20	Alive
9	LPL	Lymphoma infiltration	R-CHOP	CR	NA	63.23	Alive
10	WM	MN	CPT	NA	CR	25.83	Alive
11	LPL	AIN	CP	NA	PR	7.07	Alive
12	WM	Cryoglobulinemic GN	RCD+ Ibrutinib	PR	PR	26.13	Alive
13	WM	AL	R+ CPT	No	No	53.67	PD
14	WM	MIDD	R-CHOP	NA	No	35.00	Alive
15	WM	FSGS	NT	NA	NA	7.87	Alive
16	DLBCL	IgA nephropathy	R-CHOP	No	No	3.70	Died
17	DLBCL	Lupus nephritis	CHOP	CR	NA	14.07	Alive
18	MALT lymphoma	AL	R-CHOP	Stable	PR	61.80	Alive
19	MALT lymphoma	IgA nephropathy	R + bendamustine	CR	CR	17.17	Alive
20	MALT lymphoma	Cryoglobulinemic GN	NT	NA	NA	0.53	Died

CLL, chronic lymphocytic leukemia; WM/LPL, Waldenström macroglobulinemia/lymphoplasmacytic lymphoma; DLBCL, diffuse large B-cell lymphoma; MGUS, monoclonal gammopathy of undetermined significance; MGRS, monoclonal gammopathy of renal significance; FSGS, focal segmental glomerulosclerosis; AIN, acute interstitial nephritis; MN, membranous nephropathy; AL, light chain amyloidosis; GN, glomerulonephritis; TMA, thrombotic microangiopathy; NT, no treatment; R, rituximab; FCR, fludarabine+ cyclophosphamide+ rituximab, R-CHOP, rituximab+ cyclophosphamide+ doxorubicin+ vincristine+ prednisone; RCD, rituximab+ cyclophosphamide+ dexamethasone; CPT, cyclophosphamide + prednisone+ thalidomide; NA, not available; PR, partial response; CR, complete response; PD, peritoneal dialysis; HD, hemodialysis.

The median follow-up time was 11 months, two patients dropped out during follow-up period and four patients died. Among the patients who died, one patient with CLL died after 1 year of regular dialysis, and the other three patients died of multiple organ failure within 4 months from the diagnosis of renal disease. two patients were on regular dialysis, one of them was on peritoneal dialysis.

## Discussion

The single-center case series of 28 patients during an 11-year period reveals pathological diversity and clinical complexity of kidney disease associated with B-LPD. Previous studies have reported a limited number of cases with renal involvement in B-LPD, however, scarce data has been addressed the biopsy-proven renal pathological changes. Li et al. reviewed 20 patients with renal impairment in NHL and the most common type of lymphoma was CLL (40%), followed by DLBCL (20%) (24). However, Sun et al. (25) showed that the most common lymphoma subtype in China was DLBCL (36.2%). Inconsistent with previous studies, WM/LPL was more frequent in this study. While renal amyloidosis was one of the most common kidney pathological changes in this study (n=4), which was consistent with previous studies. Chauvee et al. found amyloidosis was the most common lesions in the study of 35 patients diagnosed of B-LPD, accounting for 31% of cases (26). A recent retrospective study conducted at the Mayo Clinic, including 57 patients with WM and other IgM-producing B-LPD, found that 47 (82%) patients had MIg-associated kidney damage, 19 (33%) of whom had amyloidosis (3). Amyloid is a heterogeneous group of diseases which is diagnosed by immunofluorescence, immunoelectron microscopy, or mass spectrometry examination, and the corresponding treatment and prognosis vary widely. It has been reported that the kidney damage associated with B-LPD was mainly AL amyloidosis, with EM examination showing randomly arranged fibrillar material ranging from 9 to 11 mm in diameter (27).

MGUS is a low-grade B-LPD manifested by monoclonal protein <3 g/dl and bone marrow plasma cells <10%. Kidney disease has been reported as a common complication of MGUS and the pathological pattern was mainly determined by the physicochemical properties of the pathogenic MIg rather than its concentration (8). In the present study, we reported a case of membranous nephropathy and two cases of AIN, representing cases of MGUS. Those biopsy-diagnosed AIN had performed immunohistochemistry examination, the results of which suggested renal infiltration were unrelated to tumor cells (**Table S1**). And other factors such as nephrotoxic drugs could be taken into account. When intrinsic nephrotoxic properties of certain monoclonal Igs or their subunits induce kidney diseases, it is referred to as MGRS which implies that the pathological consequences of MIg should take priority over the hematological status (28). The diagnosis of MGRS-related disease is established by kidney biopsy and immunofluorescence studies to identify either the monotypic immunoglobulin deposits or infers their involvement in the case of C3 glomerulonephritis or TMA with a circulating monoclonal immunoglobulin (5).

C3 glomerulonephritis is a typical MGRS-related disease primarily caused by dysregulation of the alternative complement pathway. Recent studies have reported an unusually high prevalence of MIg and TMA in patients with C3 glomerulonephritis compared to the age-matched general population (29, 30). In our study, we observed one patient with MGRS had typical renal pathology of C3 glomerulonephritis combined with TMA. Light microscopy examination showed glomerular mesangial hyperplasia, sclerosis while immunofluorescence examination showed C3 deposition without other MIg deposits. Another case of biopsy-proven, kidney-limited TMA combining with diffuse proliferative sclerosing glomerulonephritis, had coexisting paraproteins which are potentially causative in our opinion. In addition to our cases, others have reported that kidney-biopsy TMA with a clone of B-lineage cells insufficient to diagnose a malignancy, represent cases of MGRS and the presumed mechanism of which is disordered complement regulation (31–34). Previous studies suggested that MGRS strongly associated with renal prognosis, so early clone-directed intervene may be essential (7, 35). However, all three patients with MGRS in our study were untreated. The available disease-modifying treatments for MGRS are to target a monoclonal Ig-producing proliferation whose existence is frequently speculative, and therefore identifying whether kidney pathological patterns are related to paraproteinemia may shed light in designing optimal treatment strategies (28). Another major difficulty for nephrologists in clinical practice is to convince hematologists to administer timely and effective chemotherapy to patients with MGRS. It is urgent to aware the necessity of the clone-specific therapy for MGRS which need the cooperation of hematologists and nephrologists (7).

Currently, MIg-related glomerulonephritis has been widely studied, but the association between non-MIg-associated glomerulonephritis, such as FSGS, diabetic nephropathy, and B-LPD has not reached consistent results (10, 36). It has been suggested that these entities were not associated with B-LPD and may have a diagnosis consistent with hematological disease (10). Malignant lymphoma was rarely concurrent with secondary IgAN. The incidence of IgAN has been reported in association with T-cell lymphoma (37), Hodgkin’s lymphoma (38) and B-cell lymphoma in several cases (36, 39). The timing of the onset of IgAN and remission after chemotherapy suggest that IgAN may be a result of a paraneoplastic phenomenon in these cases. Studies have suggested that B-cell lymphoma may be involved in the pathogenesis of IgAN through the direct production of abnormal IgA1 and autoantibodies by dysregulated clonal B-cell populations (40). In this study, there were two cases of IgAN, diagnosed almost simultaneously with hematological disease, in which the main clinical manifestation was microhematuria rather than nephrotic-range proteinuria. One case died of multiorgan failure and the other patient reached complete response after treatment, considering that this patient had IgAN secondary to lymphoma.

It has been reported that 80% of lymphomas invading the kidney presenting as AKI, but limited studies elaborated on the type of B-cell lymphoma (41). In one study conducted by Javaugue V et al, the incidence of AKI was 56%, with the highest incidence in DLBCL (75%), and 58% and 67% for the presentation of hematuria and proteinuria respectively (10). However, in another study by Chauvet S et al. (26), hematuria (74%) and proteinuria (>50%) were found to be more frequent, whereas the incidence of AKI was only 29%. The findings of the current study were consistent with the results by Chauvet S et al. (26), with a 29% prevalence of AKI and a higher incidence of proteinuria (82%), possibly due to the similarity in the number of cases. The study by Chauvet S. et al. (26) did not include patients with DLBCL, while two patients with DLBCL were included in our study, with renal pathological changes of biopsy-proven lupus nephritis and IgAN.

Previous studies found that the most common lesions involving the kidney in BLPD were lymphoma malignant infiltration in renal tubulointerstitial, severe infiltration of tumor cells, combined with acute tubular necrosis, which may lead to severe renal involvement (10, 26). Javaugue V (8) performed an immunophenotypic analysis of renal infiltration in 50 patients with BLPD, of whom 49 patients stained positive for a B-cell marker (CD20), consistent with bone marrow immunohistochemical findings, confirming that the cells infiltrating the interstitial tissue were malignant cells. In our study, lymphocytic infiltration of interstitial tissue was observed in all 28 patients by light microscope. Further immunohistochemistry examination was performed in eight patients including five cases with biopsy-proven AIN, showing confirmed interstitial infiltration as tumor cells in three of them, with the corresponding immunophenotypes in bone marrow. The other three patients showed chronic interstitial inflammatory reaction and no lymphoma infiltration was observed. Immunohistochemistry examination is necessary to identify the type of infiltrating cells, as factors such as infection, toxicity and chemotherapeutic agents may also contribute to the development of AIN.

The study population size was too small to statistically assess the prognostic impact of chemotherapy on B-LPD related kidney damage. Three patients died within 4 months after the diagnosis of kidney disease, suggesting that the survival rates of patients with kidney diseases may be very low. However, three of the five patients who achieved hematological response developed renal response, indicating that early introduction of chemotherapy specifically targeting potential B cell clones may have the potential to prolong the survival time of patients. In this study, one patient with WM combined with cryoglobulinemic glomerulonephritis was prescribed with ibrutinib after unremarkable regimen of rituximab, cyclophosphamide and dexamethasone) (RCD). And the patient got off dialysis and achieved renal remission in a very short time. Further well-planned large prospective studies of the efficacy of ibrutinib on lymphoma concurrent with kidney disease may get more useful and reliable information.

In conclusion, the clinicopathological features of renal involvement in B-LPD are diverse and complex. MIg-associated kidney disease has been relatively well studied previously, whereas less attention has been drawed on non-MIg-associated glomerulonephritis which requires distinction from primary to secondary forms. Renal biopsy and immunohistochemistry examination have the necessity for early diagnosis and prognostic assessment. Additionally, for MGRS early and proper diagnosis is essential to maximize the chance of disease-modifying treatments targeting a monoclonal Ig-producing proliferation. Further large, randomized controlled, prospective studies are still needed to ascertain the short- and long-term efficacy of kidney lesions on MGRS.

## Data availability statement

The original contributions presented in the study are included in the article/**Supplementary Material**. Further inquiries can be directed to the corresponding authors.

## Ethics statement

The study was reviewed and approved by ethics committee of the First Affiliated Hospital of Nanjing Medical University (2022-SR-083).

## Author contributions

GN designed the research and contributed to the writing. LS analyzed the data and performed statistical analysis. CZ, YY, HM, ZW, and JL reviewed the manuscript. CX and SD conceived, coordinated the study, and had responsibility for its final content. BZ is the guarantor of this work who had complete access to all the data in the study and takes ultimate responsibility for the study design and integrity of data analysis. All authors contributed to the article and approved the submitted version.

## Funding

This work was supported by grants from the National Natural Science Foundation of China (82170699, 81870469 to YY, No. 82100767 to SD), the Natural Science Foundation of Jiangsu Province (No. BK20131030 to YY, BK20191075 to SD), the China Scholarship Council (CSC, File No. 201608320124), Chinese Society of Nephrology (17010060675 to YY, 17010090678 to SD), “PRO•Run” Fund of the Nephrology Group of CEBM(KYS2021-03-02-14 to BZ), Project of clinical capability improvement of the First Affiliated Hospital of Nanjing Medical University(to SD), the Clinic Research Center of Jiangsu Province (No. BL2014080) and the Priority Academic Program Development of Jiangsu Higher Education Institutions.

## Conflict of interest

The authors declare that the research was conducted in the absence of any commercial or financial relationships that could be construed as a potential conflict of interest.

## Publisher’s note

All claims expressed in this article are solely those of the authors and do not necessarily represent those of their affiliated organizations, or those of the publisher, the editors and the reviewers. Any product that may be evaluated in this article, or claim that may be made by its manufacturer, is not guaranteed or endorsed by the publisher.
