# Supplementary material for: Clinicopathological features and individualized treatment of kidney involvement in B-cell lymphoproliferative disorder
Source: Front Immunol. 2022 Sep 12;13:903315. doi: 10.3389/fimmu.2022.903315 (PMC9510618; doi:10.3389/fimmu.2022.903315)
Supplement: Supplementary file 1 [file Table_1.docx]

Clinicopathological features and individualized treatment of kidney involvement in B-cell lymphoproliferative disorder

**Guangyan Nie^1^, Lianqin Sun^1^, Chengning Zhang^1^, Yanggang Yuan^1^, Huijuan Mao^1^, Zhen Wang^2^，Jianyong Li^3^, Suyan Duan^1*^**^†^**，Changying Xing^1*†^, Bo Zhang^1,4*†^**

^1^Department of Nephrology, the First Affiliated Hospital of Nanjing Medical University, Nanjing Medical University, China

^2^Department of Pathology, The First Affiliated Hospital of Nanjing Medical University, Jiangsu Province Hospital, Nanjing, China

^3^Department of Hematology, The First Affiliated Hospital of Nanjing Medical University, Jiangsu Province Hospital, Nanjing, China.

^4^Department of Nephrology, Pukou Branch of Jiangsu Province Hospital (Nanjing Pukou Central Hospital), Nanjing, China.

**Background** Due to the various clinical and pathological manifestations of kidney involvement in lymphoproliferative disorder (LPD), the whole spectrum of kidney disease in LPD is still unclear, and data on kidney prognosis is scarce.

**Methods** We retrospectively reviewed the renal pathology profiles from January 2010 to December 2021, and 28 patients with B-cell LPD combined with intact renal biopsy data were included.

**Results** There were 20 men and 8 women aging 41 to 79 years at the time of renal biopsy (median age 62 years). According to hematological diagnosis, patients were classified into 4 groups: chronic lymphocytic leukemia (CLL) (group1, n=7), Waldenström macroglobulinemia/lymphoplasmacytic lymphoma (WM/LPL) (group 2, n=8; WM, n=6; LPL, n=2), Other non-Hodgkin's lymphomas (NHL) (group3, n=7; diffuse large B-cell lymphoma (DLBCL), n=2; mucosa-associated lymphoid tissue (MALT) lymphoma, n=4; Low grade B-cell lymphoma, n=1), and monoclonal gammopathy of undetermined significance/monoclonal gammopathy of renal significance (MGUS/MGRS) (group 4, n=6). Median serum creatinine (Scr) level was 129 (range,59-956) umol/L. 8 patients (29%) were presented with acute kidney injury (AKI), and 5 patients (18%) required hemodialysis upon admission. Twenty-three patients (82%) presented with proteinuria (median protein excretion, 2.14 g/d), 11(39%) of whom had the nephrotic syndrome. Interstitial malignant infiltration was the most frequent renal lesion (n=6). Eight patients underwent immunohistochemistry of renal tissues, of which 3 patients (CLL, n=1; LPL, n=1; WM, n=1) had confirmed lymphoma infiltrates, and the infiltrating cells in the remaining 5 patients (CLL, n=1; MALT lymphoma, n=2; MGUS, n=2) were considered unrelated to lymphoma. The most common glomerular diseases were renal amyloidosis (n=4) and membranous nephropathy (n=4). Only 20 patients were treated, 13 of whom were treated with rituximab separately or in combination. The median follow-up time was 11 months. Of these, 6 had achieved hematological response, complete response in 5 cases. 8 had achieved renal response. At the end-of-study visit, 4 patients died and 2 progressed to end stage kidney disease (ESKD).

**Conclusion** In conclusion, the clinicopathological spectrum of renal involvement in BLPD is diverse. Renal biopsy and immunohistochemistry are required for early diagnosis and prognostic assessment.

**Keywords: lymphoproliferative disorders, Waldenström macroglobulinemia, chronic lymphocytic leukemia, non-Hodgkin's lymphomas, monoclonal gammopathy of undetermined significance, kidney involvement**

^*^**CORRESPONDENCE**

Bo Zhang,zhangbo@jsph.org.cn;Suyan Duan,duansuyan@jsph.org.cn;Changying Xing,cyxing62@126.com

^†^These authors have contributed equally to this work and share senior authorship

Table S1 Comparison of clinicopathological findings in eight patients with immunohistochemistry of renal tissue

| **Tumor cell-associated renal infiltration** | | | | | | | | | | | | | | | | | | | | |
| --- | --- | --- | --- | --- | --- | --- | --- | --- | --- | --- | --- | --- | --- | --- | --- | --- | --- | --- | --- | --- |
| number | | | sex | | age  (year) | Types of kidney pathology | Types of Hematologic Disorders | | HB(g/L) | Lymphocyte count (*10^9/L) | | | albumin/globulin  ratio | | Proteinuria(g/d) | | Hematuria(/ul) | | Scr(umol/L) | |
| 5 | | | M | | 45 | AIN | CLL | | 102 | 10.58 | | | 2.60 | | 1.36 | | 12.0 | | 199.8 | |
| 8 | | | M | | 68 | AIN | WM | | 78 | 0.77 | | | 2.20 | | 3.10 | | 4.0 | | 182.7 | |
| 11 | | | M | | 63 | AIN | LPL | | 73 | 20.25 | | | 0.40 | | 4.53 | | 17.0 | | 233.9 | |
| **Tumor cell-unrelated renal infiltration** | | | | | | | | | | | | | | | | | | | | |
| 2 | | | M | | 75 | FSGS | CLL | | 102 | 2.55 | | | 1.60 | | 0.26 | | 968.0 | | 134.1 | |
| 18 | | | W | | 57 | AL | MALT lymphoma | | 143 | 1.69 | | | 0.63 | | 11.10 | | 13.1 | | 79.7 | |
| 21 | | | W | | 41 | FSGS | MALT  lymphoma | | 76 | 1.43 | | | 0.70 | | 8.84 | | 130.0 | | 67.6 | |
| 23 | | | W | | 55 | AIN | MGUS | | 104 | 1.70 | | | 2.30 | | 0.83 | | 2.6 | | 179.8 | |
| 25 | | | M | | 65 | AIN | MGUS | | 105 | 0.59 | | | 1.30 | | 0.20 | | 31 | | 956.0 | |
| **Clinicopathological findings** | | | | | | | | | | | | | | | | | | | | |
| number | | | urea (mmol/l) | | eGFR(mL/min/1.73 m2) | CKD stage | sC3(g/l) | | sC4(g/L) | AKI | | | Lymphadenopathy | | RK (mm) | | LK (mm) | | sMIg | |
| 5 | | | 7.39 | | 34 | 3b | 1.04 | | 0.33 | no | | | yes | | 114 | | 119 | | - | |
| 8 | | | 13.48 | | 32 | 3b | 0.58 | | 0.16 | yes | | | yes | | 106 | | 121 | | - | |
| 11 | | | 15.31 | | 25 | 4 | 0.60 | | 0.21 | yes | | | / | | / | |  | | IgG-κ | |
| 2 | | | 8.15 | | 44 | 3b | / | | / | no | | | yes | | 100 | | 98 | | - | |
| 18 | | | 2.90 | | 71 | 2 | 1.73 | | 0.35 | no | | | yes | | / | | / | |  | |
| 21 | | | 5.71 | | 97 | 1 | 0.69 | | 0.14 | no | | | no | | 116 | | 121 | | - | |
| 23 | | | 13.30 | | 27 | 4 | 0.97 | | 0.48 | yes | | | yes | | 98 | | 106 | | λ LC | |
| 25 | | | 19.75 | | 4 | 5 | 0.90 | | 0.27 | yes | | | no | | 130 | | 130 | | IgM-L | |
| **Pathologic findings** | | | | | | | | | | | | | | | | | | | | |
|  | | | | | | **Kidney tissue immunohistochemistry** | | | | | | | | | | | | | | |
| Number | | Cell type of interstitial infiltration | | | CD3 | CD5 | | | CD10 | CD19 | | CD20 | | CD23 | | CD68 | |  |  |  |
| 5 | | inflammatory cells | | | - | + | | | / | / | | + | | + | | / | |  |  |  |
| 8 | | LYMP, MON, NEUT | | | / | / | | | / | + | | + | | / | | + | |  |  |  |
| 11 | | LYMP, MON | | | - | - | | | - | / | | + | | + | | / | |  |  |  |
| 2 | | LYMP, MON | | | + | + | | | / | / | | - | | - | | / | |  |  |  |
| 18 | | LYMP, MON | | | + | + | | | - | / | | - | | - | | / | |  |  |  |
| 21 | | inflammatory cells | | | no lymphocytic infiltration | | | | | | | | | | | | |  |  |  |
| 23 | | LYMP, MON, plasma cells, atypical cells | | | + | / | | | / | + | | + | | / | | / | |  |  |  |
| 25 | | LYMP, MON, NEUT, EOSIN | | | + | + | | | - | / | | + | | + | | / | |  |  |  |

M, man; W, woman; HB, hemoglobin; SCr, serum creatinine; eGFR, estimated glomerular filtration rate; CKD, chronic kidney disease; AIN, acute interstitial nephritis; CLL, chronic lymphocytic leukemia; WM, Waldenström macroglobulinemia; LPL, lymphoplasmacytic lymphoma; MALT lymphoma, marginal zone lymphoma of mucosa-associated lymphoid tissue; FSGS, focal segmental glomerulosclerosis; AL, amyloidosis; AKI, acute kidney injury; sMIg, serum monoclonal immunoglobulin; LK, left kidney; RK, right kidney; LYMP, lymphocytes; MON, monocytes; NEUT, neutrophils; EOSIN, eosinophils; -,negative; +, positive
